# Epidemiological Characteristics and Clinical Manifestations of Hepatitis E in a Tertiary Hospital in China: A Retrospective Study

**DOI:** 10.3389/fmicb.2021.831968

**Published:** 2022-03-03

**Authors:** Li Fang, Junli Zhang, Huiying Chen, Fangfang Lv, Yunsong Yu, Xiaoxing Du

**Affiliations:** Department of Infectious Diseases, Sir Run Run Shaw Hospital, School of Medicine, Zhejiang University, Hangzhou, China

**Keywords:** hepatitis E, HEV (hepatitis E virus), elderly, fatty liver disease, IgM/IgG, HEV RNA

## Abstract

**Background:**

Hepatitis E virus (HEV) infection is the most common cause of acute viral hepatitis worldwide and one of the main causes of death in the last decade, causing chronic hepatitis and liver failure in some populations. The aging population and obesity are two major factors threatening human health. Therefore, we want to understand the relationship between these two groups and HEV infection.

**Objectives:**

The study aimed to analyze the epidemiological, clinical, and laboratory features of HEV infection and evaluate probable high-risk factors for disease progression and the current diagnostic strategies of hepatitis E infection.

**Study Design:**

Patients diagnosed with acute hepatitis E with symptoms and liver dysfunction were enrolled. For statistical analysis, clinical features and laboratory findings were collected between the elderly and non-elderly and HEV+ fatty liver disease (FLD) groups. Statistical analysis was performed using Excel and the platform VassarStats, and statistical significance was taken as *P* < 0.05.

**Results:**

Jaundice and the bilirubin peak were significantly deeper, the duration of hospitalization was significantly longer, and the proportion of ascites and liver failure was significantly higher in the elderly group. The aging population is one of the risk factors of severe hepatitis E. Hepatitis E becomes more serious in the HEV + FLD group, although the results did not reach statistical significance.

**Conclusion:**

The aging and FLD were suggested to aggravate HEV infection. However, the diagnosis of HEV infection remains a challenge. A prospective study with sufficient sample size is needed to confirm this conclusion.

## Introduction

**FIGURE 1:**
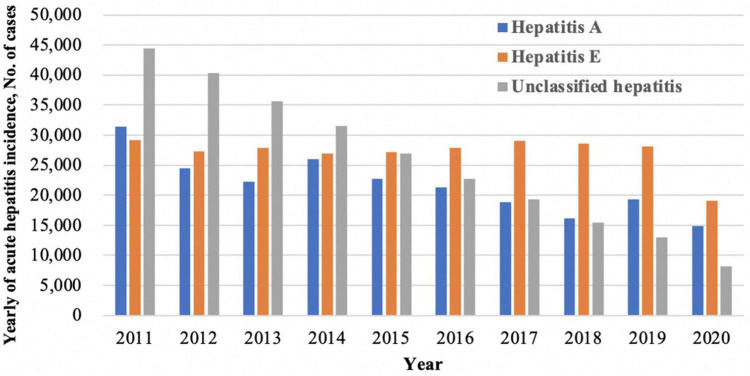
Trends in acute hepatitis cases, China, 2011–2020.

Hepatitis E virus (HEV) is the most common cause of acute viral hepatitis worldwide and represents a major global health problem (European Association for the Study of the Liver, 2018). An estimated 20 million HEV infections occur globally per year, resulting in around 70,000 deaths (Webb and Dalton, 2019). Since the first documented outbreak in 1955–1956 in India, hepatitis E has become the heaviest burden in some developing countries in Asia and Africa, where the disease is endemic and outbreaks frequently (Kmush et al., 2013; Denner et al., 2019). As one of the highest endemic areas of hepatitis E, China’s seroprevalence of anti-HEV immunoglobulin G (IgG) among the general population was 27.3% (Yue et al., 2019). According to the China Centers for Disease Control (CDC), viral hepatitis is still the leading infectious disease and one of the main causes of death in this decade. Among those acute hepatitis, hepatitis A and unclassified hepatitis showed a decreasing trend, while hepatitis E infection remained high (Figure 1).

Hepatitis E virus is a small icosahedral non-enveloped single-stranded RNA virus, classified in the genus Hepevirus, family Hepeviridae (Takahashi et al., 2010; Smith et al., 2014). It is classified into eight genotypes (HEV 1–8), of which genotypes (Gt) 1–4 can infect humans and differ in their prevalence by geographic region and route of transmission. Gt 1 and 2 infect humans waterborne, while Gt 3 and 4 are zoonotic (Doceul et al., 2016). Gt 1 and 2 have been reported mainly in Asia, India, and North Africa (Labrique et al., 2010). Gt 3 is prevalent in Western countries and Asia and North America; Gt 4 is endemic in China, Japan, and other countries in Asia (Garbuglia et al., 2013; Bouamra et al., 2014).

Hepatitis E virus infection almost causes an asymptomatic or self-limited acute infection. However, accumulating literature has reported that hepatitis E may increase the risk of chronic infection and induce acute liver failure in some special populations. As reported, acute HEV infection during pregnancy has been associated with a 15–25% mortality rate (Kane et al., 1984; Patra et al., 2007). Immunocompromised patients are especially at risk for developing chronic hepatitis, resulting in progressive liver disease, even cirrhosis (Dalton et al., 2009; Kamar et al., 2011; Legrand-Abravanel et al., 2011; Kmush et al., 2015), causing significant morbidity and mortality (Tseng et al., 2020). Infection with HEV can lead to hepatic decompensation in patients with pre-existing liver disease (Hamid et al., 2002). The impact of hepatitis E infection on pregnant women and immunocompromised hosts cannot be ignored.

Hepatitis E virus infections are not limited to the liver but may also affect other organs, including thrombocytopenia, hemolysis, acute thyroiditis, membranous glomerulonephritis, acute pancreatitis, neurologic diseases like meningoencephalitis, Guillain–Barré syndrome, and so forth (Geurtsvankessel et al., 2013). Some have no liver injury, which leads to a difficult diagnosis of acute hepatitis E infection (Pischke et al., 2017).

The diagnosis of HEV infection is complicated by a lack of a standardized assay, which is based primarily on clinical, biochemical, and radiologic findings, anti-HEV-antibodies (IgM, IgG, or both), HEV antigen detected by enzyme immunoassays, and HEV RNA detected using PCR; even HEV ORF2 protein can be used for a histopathologic diagnosis of hepatitis E by immunohistochemistry (European Association for the Study of the Liver, 2018). However, both false positive and false negative are common in serological tests for hepatitis E virus, and according to the serological and virological courses of the virus itself (Aggarwal, 2013b), anti-HEV-IgM is not produced in the early onset, and anti-HEV IgG may remain detectable for many years. In some immunocompromised patients with HEV infection, anti-HEV antibodies remained unproducted (Dalton et al., 2009). Molecular diagnoses of HEV RNA are highly recommended. However, RT-PCR is relatively expensive and technically challenging, limiting its accessibility. The accurate diagnosis of hepatitis E is still challenging.

Our study aimed to analyze the epidemiological, clinical, and laboratory features of HEV infection, evaluate probable high-risk factors for disease progression, and then briefly discuss which diagnostic test is most accurate to detect HEV infection in a real-world setting.

## Patients and Methods

**FIGURE 2:**
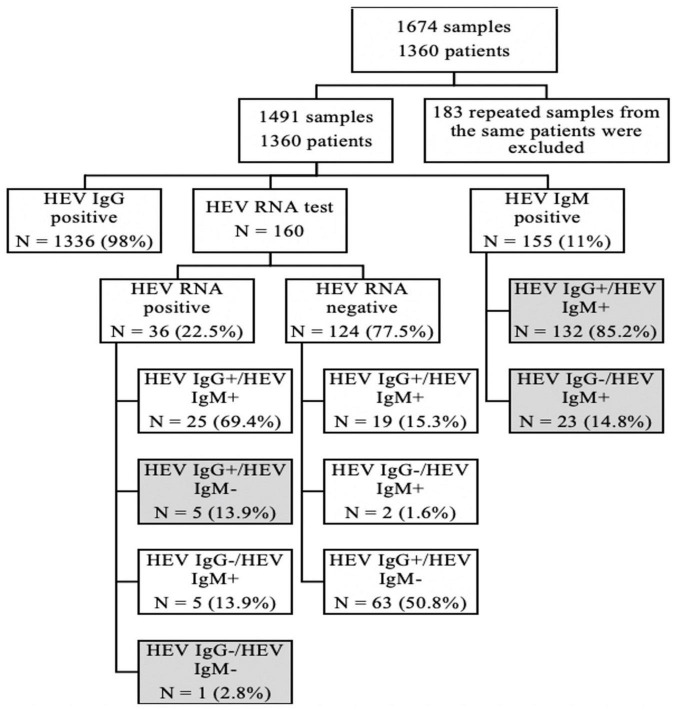
Our patient cohort was stratified according to HEV serology and PCR results.

We screened out samples, which were positive for the anti-HEV-IgM or anti-HEV-IgG test through the case system in the Sir Run Run Shaw Hospital, School of Medicine, Zhejiang University, from January 2020 to October 2021, and a total of 1,674 samples were obtained. One hundred eighty-three repeated test results of the same patient from the different times were excluded, and only the first positive sample was retained for study. Finally, 1,491 samples involving 1,360 patients were retained for a further analysis. Among those patients, only 160 patients were detected with HEV RNA for the lack of the test item in our hospital. Patients were divided into groups based on HEV serology and PCR results (Figure 2).

**FIGURE 3:**
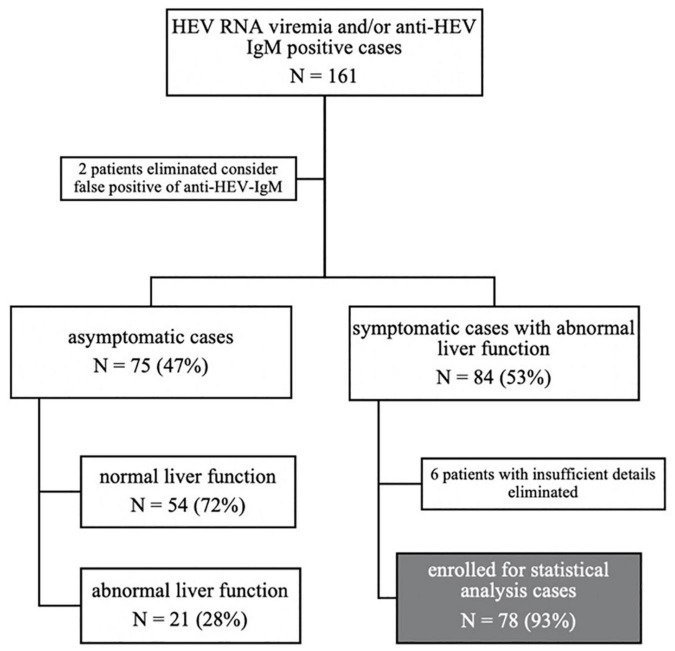
Flow diagram: study participants were grouped according to symptoms, liver function, and the final enrolled populations for our study.

Acute HEV infection was defined by ALT/AST elevation and a positive HEV PCR test or positive anti-HEV IgM serology, with clinical symptoms, and a suspected diagnosis of acute HEV infection was defined by anti-HEV IgM positive with or without ALT/AST elevation, but no clinical symptoms. We identified 161 patients with either anti-HEV-IgM or HEV RNA who were positive in both outpatient and inpatient departments, and two patients were eliminated to consider false positives of anti-HEV-IgM. One hundred fifty-nine patients were enrolled for demographic analysis. Seventy-eight patients diagnosed with defined acute HEV infection were enrolled for statistical analysis after excluding six patients with insufficient details (Figure 3).

As population aging and obesity are two major factors threatening human health, patients were divided into the elderly group and the non-elderly group based on the age of 60. Clinical features, laboratory findings, and prognosis were compared between these two groups. Similarly, patients were divided into HEV group and HEV + FLD group, according to whether FLD was combined or not. Clinical features, laboratory findings, and prognosis were collected for statistical analysis. The finding of fatty liver mainly depends on imaging, such as ultrasound, CT scan, or MRI. The diagnostic performance of the anti-HEV-antibodies and HEV RNA detection was briefly discussed in the study.

All enrolled patients gave informed consent as approved by the Research Ethics Committee of the Sir Run Run Shaw Hospital, School of Medicine, Zhejiang University.

All serum samples were analyzed for anti-hepatitis E virus (HEV) enzyme-linked immunosorbent assay (ELISA) with the HEV IgM/HEV IgG test, developed by Wantai BioPharm, Beijing, China, according to the hospital laboratory’s description. According to the manufacturer’s instructions, the level of HEV RNA was detected using quantitative real-time reverse transcription-polymerase chain reaction (RT-qPCR) (TIANamp virus RNA kit, Tiangen, Beijing, China).

Statistical analysis was performed using Excel V16.56.21121100 and the platform VassarStats, and *P* < 0.05 was considered a statistically significant difference. Quantitative variables are presented as mean ± standard deviation (SD) or as medians and range. Qualitative variables are presented as frequencies and ratios (percent). The χ^2^ and Fisher’s exact tests were applied to assess associations between two qualitative variables. The comparison of quantitative variables between two independent groups was carried out using the two-sample *t*-test or the non-parametric Mann–Whitney test.

## Results

**FIGURE 4:**
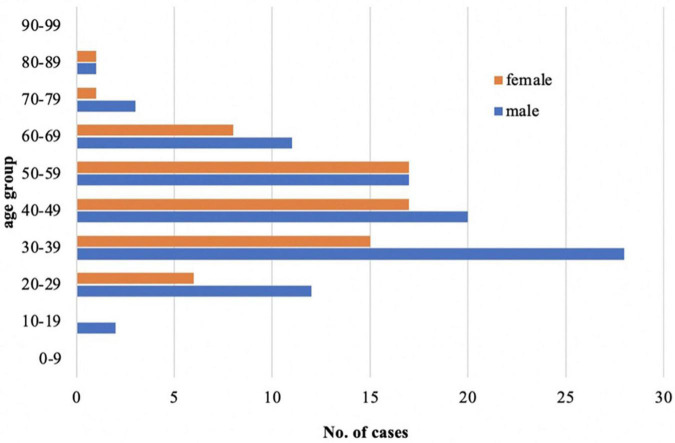
Demographic characteristics of the study population: comparing the incidence of acute hepatitis E infection between males and females in different age stages.

**FIGURE 5:**
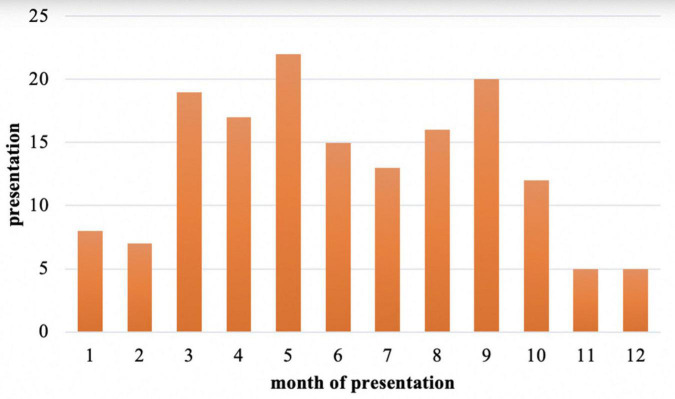
The monthly incidence of acute hepatitis E infection from January 2020 to October 2021.

Among those 159 cases with a defined or suspected diagnosis of acute HEV infection, the demographic characteristics of the populations were analyzed: 94 males (59%) and 65 females (41%), with a male to female ratio of 1.45:1 (Figure 4). Males were significantly higher than females (χ^2^ = 21.405, *P* < 0.05). The median age was 42 (range 18–84) years. Hepatitis E is sporadic throughout the year, more in March, May, and September (Figure 5), but the results may be inaccurate due to the data lacking from November to December 2021.

Among those 159 cases, 81 (51%) were from the outpatient system, while 78 (49%) were from the inpatient system. Seventy-five (47%) asymptomatic cases were detected by routine physical examination, with 54 (72%) having a normal liver function and 21 (28%) abnormal liver function, whose jaundice was all negative. Symptoms occurred in 84 (53%) cases. All of them had abnormal liver function. Among those symptomatic patients, acute icteric hepatitis accounts for 70 (83%) and non-icteric hepatitis 14 (17%). Six cases were not enrolled due to incomplete data. Finally, 78 cases with symptoms and abnormal liver function were enrolled for statistical analysis. The details of these included studies are shown in Figure 3.

The baseline demographics and clinical characteristics of the enrolled patients are shown in Table 1. Among the total of 78 patients, 53 (68%) patients were male and 25 (32%) patients were female, with a median age of 46 (range 20–84), and 24 (31%) patients were more than 60 years old. Body mass index (BMI) [kg/m^2^, cut-points established by the National Health and Family Planning Commission of China (NHFPC) for Chinese adults (China NHaFPCo, 2013)]: underweight, <18.5 kg/m^2^, normal weight, 18.5–23.9 kg/m^2^, overweight but not obese, 24.0–27.9 kg/m^2^, and obese ≥28.0 kg/m^2^. Among them, 47 (60%) patients were normal weight, with 5 patients (6%) underweight, 19 (24%) patients overweight, and 7 (9%) patients obese.

**TABLE 1:** Demographics and clinical characteristics in 78 patients with acute HEV infection.

Variables		Number of patients	%
Sex	Male	53	68
	Female	25	32
Age	Median (range year)	46 (20–84)	–
	≥ 60	19	24
	<60	59	76
BMI	Underweight	5	6
	Normal weight	47	60
	Overweight	19	24
	Obese	7	9
Alcohol drinking		31	40
Pregnancy		3	4
Family history of liver disease		12	15
Chronic hepatitis B		11	14
Fatty liver		39	50
Alcoholic liver disease		18	23
Cirrhosis		12	15
Clinical manifestations	Malaise	67	86
	Anorexia	60	77
	Muscle pain	20	26
	Nausea/vomiting	43	55
	Abdominal pain	40	51
	Jaundice	57	73
	Fever	29	37
	Diarrhea	13	17
	Pruritus	12	15
Ascites		9	12
Hepatic encephalopathy		0	0
Duration of hospitalization	≥ 14 days	14	18
	<14 days	64	82
Prognosis	Improved	76	97
	Progressed	2	3

In our study, the main symptoms of hepatitis E were malaise (86%), anorexia (77%), nausea/vomiting (55%), abdominal uncomfortable (51%), jaundice (73%), and fever (37%). The established clinical and physical data are presented in Table 1. Some patients co-existed chronic liver diseases, including alcoholic liver disease 18 (23%), fatty liver disease 39 (50%), chronic hepatitis B infection 11 (14%), and liver cirrhosis 12 (15%). Eight (10%) developed severe hepatitis, and 2 (3%) progressed at discharge. Fortunately, in 3 (4%) pregnant women, none of them developed liver failure during disease. Most of the patients recovered quickly, 64 (82%) patients were discharged within 14 days or even less than 7 days, and only 14 (18%) patients were hospitalized for more than 2 weeks. Ascites occurred in 9 (12%) patients, and no hepatic encephalopathy happened. Eight (10%) patients developed liver failure, and 2 (3%) patients progressed during discharge. The details of these included studies are shown in Table 1.

Patient demographics, clinical characteristics, and laboratory results were compared between the elderly group (≥60-year-old) and the non-elderly group (<60-year-old) in Table 2. The elderly group had statistically significantly higher symptoms of abdominal pain (*P* = 0.005) and diarrhea (*P* = 0.002) compared with the non-elderly group. The peak levels of ALT, AST, r-GT, AKP, TBIL, DBIL, TBA, coagulation function (INR), albumin, globulin levels during the disease, and CBC on the first day of visit were selected. As a result, HGB (*P* = 0.016) and ALT (*P* = 0.008) levels were significantly lower in the elderly group, while TBIL/DBIL levels were statistically significantly higher in the non-elderly group (all *P* ≤ 0.001).

**TABLE 2:** Patients demographics, clinical characteristics, and laboratory findings between the elderly group (≥60-year-old) and the non-elderly group (<60-year-old).

Clinical features	≥ 60 (*n* = 19, %)	<60 (*n* = 59, %)	*P* value
Male	12 (63)	41 (69)	0.402
Normal BMI	13 (68)	34 (58)	0.288
Abnormal BMI	6 (32)	25 (42)	0.288
Alcohol drinking	10 (53)	21 (36)	0.147
Chronic hepatitis B	3 (16)	8 (14)	0.534
Fatty liver	9 (47)	30 (51)	0.500
Alcoholic liver disease	6 (32)	12 (20)	0.238
Cirrhosis	6 (32)	2 (3)	**0.002**
**Symptoms**			
Malaise	18 (95)	49 (83)	0.189
Anorexia	15 (79)	45 (76)	0.540
Muscle pain	6 (32)	14 (24)	0.344
Nausea/vomiting	12 (63)	31 (53)	0.295
Abdominal pain	15 (79)	25 (42)	**0.005**
Jaundice	16 (84)	41 (69)	0.169
Fever	7 (37)	22 (37)	0.598
Diarrhea	8 (42)	5 (8)	**0.002**
Pruritus	5 (26)	7 (12)	0.126
Ascites	6 (32)	3 (5)	**0.005**
Liver failure	4 (21)	4 (7)	0.093
Icteric hepatitis	19 (100)	38 (64)	**0.001**
**Laboratory parameters**	**Mean ± SD**	**Mean ± SD**	***P* value**
WBC (3.5–9.5 × 10^9^/L)	6.71 (3.21)	5.48 (2.95)	0.070
HGB (130–175 g/L)	135.47 (17.37)	149.31 (20.77)	**0.016**
PLT (125–350 × 10^9^/L)	159 (64.60)	131.69 (38.25)	0.375
Neutrophil (1.8–6.3 × 10^9^/L)	4.24 (2.76)	3.37 (2.21)	0.097
Lymphocyte (1.1–3.2 × 10^9^/L)	1.63 (1.10)	1.55 (1.11)	0.409
INR (0.9–1.1)	1.28 (0.62)	1.35 (0.80)	0.394
CRP (<6 mg/L)	19.45 (27.24)	9.75 (5.46)	0.272
ALT (9–50 U/L)	1084.63 (1100.36)	2493.23 (1664.04)	**0.008**
AST (15–40 U/L)	916.79 (938.72)	2226.92 (1760.40)	0.074
r-GT (10–60 U/L)	208.84 (146.45)	245.69 (149.97)	0.065
AKP (45–125 U/L)	236.58 (86.79)	192.46 (74.14)	0.327
TBIL (0–26 μmol/L)	256.34 (162.45)	73.63 (70.88)	**<0.001**
DBIL (0–4 μmol/L)	170.68 (117.14)	41.08 (38.90)	**0.001**
Albumin (40–55 g/L)	31.26 (5.24)	39.05 (3.96)	0.191
Globulin (25–35 g/L)	27.84 (6.68)	26.18 (4.88)	0.285
TBA (0–15 μmol/L)	291.08 (155.04)	217.50 (234.63)	0.405
Duration of hospitalization [mean (range day)]	15.28 (5–48)	10.78 (3–50)	**0.047**

*All the categorical variables were expressed as numbers (%). Quantitative variables were presented as mean ± standard deviation (SD) or as medians and range. P > 0.05 indicates no significant difference detected between the variances of the two samples. In the column of laboratory parameters, numbers in parentheses are the reference range of test indicators in our hospital. ALT, alanine aminotransferase; AST, aspartate aminotransferase; r-GT, glutamyl transpeptidase; AKP, alkaline phosphatase; TBIL, total bilirubin; DBIL, direct bilirubin; TBA, total bile acid; INR, international normalized ratio; CRP, C-reactive protein; WBC, white blood cell; HGB, hemoglobin; PLT, platelet. The bold values means p < 0.05, with a statistical significance.*

The high proportion of ascites in the elderly group may be related to more patients with cirrhosis, but all patients in the elderly group 19 (100%) have jaundice, while the proportion of icteric hepatitis in the non-elderly group 38 (64%) was significantly lower (*P* = 0.001). At the same time, the average duration of hospitalization in the elderly group, 15.28 days, was significantly longer than that in the non-elderly group, 10.78 days (*P* = 0.0047).

As FLD is also a worldwide public health problem, it accounts for a large proportion of 39 (50%) of our enrolled patients. Thus, patient’s demographics, clinical features, and laboratory results were also compared between the HEV group and the HEV + FLD group in Table 3. Thirty-two (82%) cases of HEV/FLD group were male, and most of them drank alcohol 24 (62%); male, drinking history, abnormal BMI, and patients with alcoholic liver disease were all statistically significantly higher, compared with the simple HEV group (*P* < 0.05). Patients in the HEV + FLD group were more prone to fatigue (*P* = 0.024). The HGB and neutrophil levels were statistically significantly higher in the HEV + FLD group. Furthermore, the peak level of r-GT was statistically significantly higher in the HEV + FLD group. The levels of transaminase, jaundice, bile acid, icteric hepatitis, ascites, and liver failure were higher in the HEV + FLD group, although there was no significant difference. At the same time, the average duration of hospitalization in the HEV + FLD group was 12.31 days, which was longer than that in the simple HEV group (9.95 days).

**TABLE 3:** Patients demographics, clinical characteristics, and laboratory findings between HEV group and HEV + FLD group.

Clinical features	HEV (*n* = 39, %)	HEV + FLD (*n* = 39, %)	*P* value
Male	21 (54)	32 (82)	**0.007**
Age [median (range year)]	42 (20–84)	49 (30–77)	0.468
Alcohol drinking	7 (18)	24 (62)	**<0.001**
Abnormal BMI	10 (26)	21 (54)	**0.001**
HEV + HBV	5 (13)	6 (15)	0.499
HEV + ALD	1 (3)	17 (44)	**<0.001**
Cirrhosis	2 (5)	6 (15)	0.131
**Symptoms**			
Malaise	30 (77)	37 (95)	**0.024**
Anorexia	26 (67)	33 (85)	0.056
Muscle pain	10 (26)	10 (26)	0.500
Nausea/vomiting	21 (54)	22 (56)	0.500
Abdominal pain	18 (46)	22 (56)	0.249
Jaundice	27 (69)	30 (77)	0.305
Fever	18 (46)	11 (28)	0.079
Diarrhea	6 (15)	7 (18)	0.499
Pruritus	7 (18)	5 (13)	0.377
Ascites	4 (54)	5 (54)	0.500
Liver failure	3 (54)	5 (54)	0.356
Icteric hepatitis	32 (54)	35 (54)	0.259
**Laboratory parameters**	**Mean ± SD**	**Mean ± SD**	***P* value**
WBC (3.5–9.5 × 10^9^/L)	5.44 (2.29)	6.38 (2.97)	0.064
HGB (130–175 g/L)	138.23 (20.93)	149.89 (17.48)	**0.005**
PLT (125–350 × 10^9^/L)	158.69 (51.76)	152.23 (57.62)	0.306
Neutrophil (1.8–6.3 × 10^9^/L)	3.12 (1.40)	4.22 (2.63)	**0.013**
Lymphocyte (1.1–3.2 × 10^9^/L)	1.68 (1.21)	1.49 (0.80)	0.207
INR (0.9–1.1)	1.43 (1.60)	1.27 (0.65)	0.289
CRP (<6 mg/L)	12.16 (21.04)	18.56 (42.29)	0.202
ALT (9–50 U/L)	1741.13 (1346.09)	1930.69 (1754.50)	0.299
AST (15–40 U/L)	1275.15 (1086.97)	1329.18 (1532.61)	0.429
r-GT (10–60 U/L)	239.18 (179.99)	360.31 (370.90)	**0.037**
AKP (45–125 U/L)	230.82 (112.47)	222.66 (102.15)	0.371
TBIL (0–26 μmol/L)	158.04 (153.51)	160.63 (133.94)	0.468
DBIL (0–4 μmol/L)	100.10 (106.10)	106.99 (114.11)	0.394
Albumin (40–55 g/L)	35.51 (5.98)	36.23 (5.86)	0.302
Globulin (25–35 g/L)	27.29 (5.81)	26.59 (5.88)	0.191
TBA (0–15 μmol/L)	277.87 (213.93)	282.96 (224.04)	0.460
Duration of hospitalization [mean (range day)]	9.95 (3–39)	12.31 (3–50)	0.121

*The bold values means p < 0.05, with a statistical significance.*

Among 160 patients, we did both HEV RNA and HEV IgM/HEV IgG test, and there were 36 (22.5%) samples who were HEV RNA positive. Among them, 5 (14%) cases were HEV IgM negative and HEV IgG positive, and 1 (3%) case was both HEV IgM and IgG negative.

## Discussion

Hepatitis E virus infection is a public health concern and is the most common cause of acute viral hepatitis worldwide (Hoofnagle et al., 2012; Denner et al., 2019). Hepatitis E is mainly asymptomatic or mildly symptomatic (Zhu et al., 2010) and generally causes an acute self-limited infection. We showed that 47% of patients were asymptomatic in our study, only detected by routine physical examination. Also, anti-HEV IgM positivity could not represent present infection, and as it can last for 6 months or even longer, we also reviewed that these asymptomatic suspected HEV infections had not been seen or hospitalized due to liver disease in the past 6 months. HEV infection is sporadic throughout the year. In our study, most patients were male, with a male to female ratio of 1.45:1, and an age range of onset mainly from 30 to 60 years old, as literature has reported that HEV infection mainly affects middle-aged and elderly men (Lu et al., 2006). Our study found that all the asymptomatic HEV infections were negative of jaundice. Even with high elevated transaminase, it is considered that jaundice might be an important indicator to assess whether symptoms occurred and the severity of the disease. HEV infection caused by Gt 1 and 2 occurred most frequently in older children and young adults, while Gt 3 and 4 occurred in much older age (Purcell and Emerson, 2008). Gt 4 is the dominant genotype of sporadic HEV infection in China. As the aging of the population is becoming more and more serious, the elderly tend to have a variety of chronic diseases, such as diabetes, hypertension, heart disease, and neoplastic diseases. Therefore, we need to pay more attention to the hepatitis E infection in the elderly.

In the current study, the proportion of elderly patients (≥60-year-old) was 24% (19 out of 78 patients). People infected with acute hepatitis E often feel fatigued, have anorexia and jaundice, and are usually accompanied by nausea, vomiting, abdominal pain, fever, and hepatomegaly (Harun-Or-Rashid et al., 2013), and some relatively rare features like diarrhea, pruritus, and urticarial rash. As in our study, the elderly seemed to have a higher chance of developing symptoms than non-elderly patients. All patients in the elderly group suffered jaundice, and the peak of bilirubin was significantly deeper, the duration of hospitalization was significantly longer, and the proportion of ascites and liver failure was significantly higher. Therefore, age is one of the risk factors of severe hepatitis E. We need to pay more attention to the elderly with hepatitis E infection.

Infection with HEV can lead to hepatic decompensation in patients with pre-existing liver disease (Hamid et al., 2002; Chow et al., 2014). Patients with alcoholic liver disease have clinically severe disease with high mortality when exposed to HEV (Dalton et al., 2011). Previous studies mainly focused on viral hepatitis, especially hepatitis B infection. Currently, the incidence of NAFLD has been increasing dramatically over time. It is estimated that NAFLD prevalence is approximately 27.4% in Asia (Lonardo et al., 2016). Fifty percent of patients enrolled in our study were accompanied by FLD. Therefore, we also need to know the effect of fatty liver disease in hepatitis E. Study shows that most of them were male, with alcohol drinking and existing malnutrition or obesity. Our study showed that the severity of the disease was higher in the HEV + FLD group. FLD may aggravate the HEV infection, although there was no significant difference.

Among the 160 samples tested for HEV RNA, we found HEV RNA positive cases, while HEV IgM was negative or both HEV IgM/IgG were negative. It is not enough to only test HEV IgM/IgG or HEV RNA, leading to a missed diagnosis. As we know, most of the patients infected with hepatitis E are asymptomatic or even have no biochemical changes, and they do not go for a physical examination. Therefore, many cases may be unrecognized or misdiagnosed, and the actual number of acute hepatitis E infections in the population may be much more than reported. Seroprevalence studies suggest that as much as one-third of the world’s population will be infected at some point during their lifetime (Purcell and Emerson, 2005). Dalton et al. suggested that diagnosis of drug-induced liver injury is not secure without testing for HEV (Dalton et al., 2007). Studies show that non-specific autoantibodies associated with autoimmune hepatitis (AIH) are frequently present during acute HEV infection (Terziroli Beretta-Piccoli et al., 2018), and the prevalence of anti-HEV IgG in patients with AIH was found to be higher than that in the general local population (van Gerven et al., 2016; Eder et al., 2019); a false positive HEV serologies in AIH have been reported (Valota et al., 2019). Immunosuppression can lead to chronic HEV disease (de Niet et al., 2012; Schlosser et al., 2012). Clinicians should differ acute HEV infection from AIH.

To accurately diagnose acute hepatitis E infection, we need to combine the patient’s clinical symptoms, liver function, hepatitis E serology, and HEV RNA. Unfortunately, both serologic and molecular tests vary greatly in sensitivity and specificity, and false positives and negatives are common (Aggarwal, 2013a). The timing of the appearance of HEV markers will also affect the test results. HEV RNA does not last long and cannot be detected in the blood about 3 weeks after onset of symptoms. The virus continued to spread in feces for a further 2 weeks (Souza et al., 2019). HEV IgM appears early and disappears over 4–5 months (Favorov et al., 1992). However, we found that several HEV IgM patients persisted positive for more than 1 year among our patients. HEV IgG is long-lasting and is estimated to persist for up to 80 years after a natural infection (Chadha et al., 1999). Some researchers want to develop a multifactor model to improve the diagnosis of HEV infection in resource-limited settings (Lu et al., 2021). However, the diagnostic methods for hepatitis E clinically are still very limited.

## Conclusion

In summary, acute HEV infection mainly affects middle-aged and older men and most patients appear asymptomatic or mildly symptomatic in this retrospective study. Acute hepatitis E can be chronic and can even progress to liver failure and decompensated cirrhosis in some special populations. Aging was one of the risks that were correlated with the severity of HEV infection. Patients complicated with FLD resulted in higher liver injury and related complications, although there was no statistically significant difference. The diagnosis of acute HEV infection remains a challenge due to the limitation of testing time and the false-positive and false-negative problem. An accurate method for identifying active, recent, and past HEV infections is urgent.

The limitations of our study are the retrospective character and the relative bias due to the recruitment of patients from one tertiary hospital. A prospective study with a sufficient sample size would be needed for further analysis.

## Data Availability Statement

The original contributions presented in the study are included in the article/supplementary material, further inquiries can be directed to the corresponding author/s.

## Ethics Statement

The studies involving human participants were reviewed and approved by the Sir Run Run Shaw Hospital, School of Medicine, Zhejiang University. The patients/participants provided their written informed consent to participate in this study.

## Author Contributions

LF and XD conceived and designed the study. JZ and HC collected the clinical data. FL and YY conducted the analysis and coordinated the management of the study. LF drafted the manuscript. XD discussed the scientific issues of the study and helped to draft the manuscript. All authors read and approved the final version of the manuscript.

## Conflict of Interest

The authors declare that the research was conducted in the absence of any commercial or financial relationships that could be construed as a potential conflict of interest.

## Publisher’s Note

All claims expressed in this article are solely those of the authors and do not necessarily represent those of their affiliated organizations, or those of the publisher, the editors and the reviewers. Any product that may be evaluated in this article, or claim that may be made by its manufacturer, is not guaranteed or endorsed by the publisher.
